# Reliability of Using Retinal Vascular Fractal Dimension as a Biomarker in the Diabetic Retinopathy Detection

**DOI:** 10.1155/2016/6259047

**Published:** 2016-09-14

**Authors:** Fan Huang, Behdad Dashtbozorg, Jiong Zhang, Erik Bekkers, Samaneh Abbasi-Sureshjani, Tos T. J. M. Berendschot, Bart M. ter Haar Romeny

**Affiliations:** ^1^Department of Biomedical Engineering, Eindhoven University of Technology, Eindhoven, Netherlands; ^2^University Eye Clinic Maastricht, Maastricht, Netherlands; ^3^Department of Biomedical and Information Engineering, Northeastern University, Shenyang, China

## Abstract

The retinal fractal dimension (FD) is a measure of vasculature branching pattern complexity. FD has been considered as a potential biomarker for the detection of several diseases like diabetes and hypertension. However, conflicting findings were found in the reported literature regarding the association between this biomarker and diseases. In this paper, we examine the stability of the FD measurement with respect to (1) different vessel annotations obtained from human observers, (2) automatic segmentation methods, (3) various regions of interest, (4) accuracy of vessel segmentation methods, and (5) different imaging modalities. Our results demonstrate that the relative errors for the measurement of FD are significant and FD varies considerably according to the image quality, modality, and the technique used for measuring it. Automated and semiautomated methods for the measurement of FD are not stable enough, which makes FD a deceptive biomarker in quantitative clinical applications.

## 1. Introduction

The blood vessels, as part of the human circulatory system, transport the blood with nutrition and oxygen and remove the waste throughout the body. The development of the vascular system is not a random process but follows a set of optimization principles, such as the minimum friction between the blood flow and the vessel wall, the optimal heart rate to achieve proper blood supply, and the shortest transportation distance [1]. In many diseases such as diabetes, glaucoma, hypertension, and other cardiovascular diseases, these optimal conditions are no longer maintained, leading topological abnormalities to appear in the vascular network. Vessels in organs like the brain, the lung, or the kidney can only be observed indirectly by certain image modalities, such as magnetic resonance angiograph, CTA, and X-ray angiography. However, the vasculature in the nerve fiber layer of the retina can be observed directly and noninvasively by fundus cameras. Therefore, increasing attention has been drawn to the retinal images for the quantitative analysis of retinal blood vessels, which might provide useful information about the progress of systemic and cardiovascular diseases.

One of the biomarkers that could describe changes in microvasculature due to the disease progression is the fractal dimension (FD). The theory of FD was first introduced by Mandelbrot in 1983 [2]. He proposed a set of mathematical definitions for a self-similar object and used a noninteger number to describe the dimension of this highly irregular shape. In 1989, the fractal dimension was first introduced into the ophthalmology by Family et al. [3]. After that, there has been a growing interest in studying the association between the fractal dimension of the retinal vasculature and the disease severity and progression [4–8].

In many clinical studies, the fractal dimension has shown its potential in characterizing the growth of neurons, tissues, and vessels. Firstly, the fractal describes growing progression of the neuron cells by quantifying their complex dendrites. For instance, Ristanović et al. [9] and Milošević et al. [10] studied the morphology of the branching patterns in the cortical neuronal dendrites by fractal dimension and Reichenbach et al. [11] used it as a discriminator for different mammalian astroglial cell types. In the case of tissue image analysis, Li et al. [12] applied the fractal calculation on medical tissue images in order to detect the special texture of pathological tissues. In addition, fractal dimension was used as a feature for parenchymal lung disease detection [13]. Finally, as is the focus of this paper, the fractal dimensions have been applied widely on human retinal images for disease detection.

However, we found conflicting findings in different clinical studies. Some literature reports a higher FD in images of a patient group with a late stage of proliferative diabetic retinopathy compared to a healthy control group [4, 8, 14]. Broe et al. [7] did a fractal analysis on optic disc centered images of 180 patients who had type 1 diabetes in a 16-year follow-up study. They compared the fractal dimension of the patients with their values that were recorded 16 years ago and found that the fractal dimension was generally decreased. Similarly, Grauslund et al. [6] compared the box dimension of 94 type 1 diabetes patients without proliferative retinopathy to 79 patients with proliferative retinopathy (PR). They found that the PR group had lower dimension than the group without PR. Also papers report mixed results when comparing healthy and diabetic groups. In the study of Aliahmad et al. [8], 189 optic disc centered retinal images of healthy and diabetic individuals were examined with box dimension. The statistical results showed that the healthy subjects had higher fractal dimension than the diabetic group. However, Yau et al. [5] found higher fractal dimensions in the diabetic group with 498 patients compared to those in the normal group with 743 healthy subjects. Moreover, the cross-sectional study conducted by Cheung et al. [4] showed that the longer the diabetic duration of one patient was, the higher his retinal fractal dimension was.

Of course, all the above-mentioned studies had different setups. Not only the number of patients but also the cameras used in data acquisition in each study were different. Therefore, the images' resolution, illumination, and quality varied across studies. Moreover, the computer software which semiautomatically does the optic disc detection, vessel segmentation, vessel skeletonization, and the fractal computation was also different in each study. Finally, the region of interest for FD calculation was not the same for all studies. These different experimental settings, therefore, may be the reasons of conflicting findings in each study.

In that case, it is worth to investigate the reliability of the FD measurement, since the measurement itself might not be stable enough to provide reliable results. Previously, few works analyzed the stability and the reliability of FD measurements. Wainwright et al. [14] studied the robustness of the FD measurement in terms of variation of brightness, focus, contrast, and image format and concluded that FD is highly sensitive to all these factors. MacGillivray and Patton [15] reported that the segmentation threshold value significantly affected the FD. Mendonça et al. [16] found that the FD was highly dependent on both vessel segmentation and FD calculation methods.

In our previous study [17], we have examined the stability of multiple fractal measurements in different cases. In this paper, we extend the previous work into 6 cases, in which we calculated the variation of the fractal dimension. (1) We calculated the FD values in groups of subjects with various diabetic retinopathy grades, where the intergroup and intragroup variations are compared. (2) We calculated the fractal dimension on the manual vessel segmentation annotated by different human observers. (3) We investigated the stability of FD using different vessel segmentation methods. (4) We explored the changes of FD in various regions of interest. (5) We tuned the segmentation threshold values to examine the influence of segmentation accuracy on the fractal measurements and (6) we compared the fractal dimensions that are calculated on the images acquired by different cameras.

The paper is organized as follows: in Section 2, we introduce the materials and datasets used in this study. In Section 3, we explain the pipeline for computing the fractal dimension, including four state-of-the-art vessel segmentation methods, the region of interest determination, and three classic fractal dimension calculation methods that are widely used in clinical studies. In Section 4 the results of different cases are compared, and the discussion is presented in Section 5. Finally, Section 6 summarizes the conclusions.

## 2. Materials

In this section, we introduce the public retinal image datasets and the test image dataset that were used in the stability studies. We used three datasets: MESSIDOR, DRIVE, and a test dataset including images captured by five different cameras.

### 2.1. MESSIDOR Database

The MESSIDOR public dataset [21] includes 1200 eye color fundus images with diabetic retinopathy grades (R0, R1, R2, and R3). The grades are provided based on the number of microaneurysms and hemorrhages and the presence of neovascularization. The images were taken in 3 ophthalmologic departments in France by using the Topcon TRC NW6 (Topcon, Japan) with field of view (FOV) of 45 degree. The images have three different sizes: 1440 × 960, 2240 × 1488, and 2304 × 1536. In this paper, we use this dataset for investigating the intragroup FD differences.

### 2.2. DRIVE Database

The DRIVE dataset [22] contains 40 fovea centered color retinal images, which were captured on 33 nondiabetic retinopathy subjects and 7 with mild early diabetic retinopathy. The images were acquired by a Canon CR5 nonmydriatic 3CCD camera (Canon, Japan) with a FOV of 45 degree. The 40 images were randomly divided into a training set and a testing set of equal size. In the testing set, the images were manually annotated by 2 different well-trained ophthalmologists. These 20 test images were used for the fractal stability and robustness study.

### 2.3. 5 Cameras Dataset

In order to investigate the variation of FD computed on the images acquired by different cameras, we established a new dataset which consists of the retinal images captured by 5 different fundus cameras on 12 young healthy volunteers. The 5 fundus cameras were installed in the Ophthalmology Department of the Academic Hospital Maastricht (AZM) in Netherlands. The volunteers are young students with 20 to 25 years of age. The retinal photographs were taken on the left eye of every subject 5 times with each camera, both fovea centered and optic disc centered (120 images in total).

The 5 cameras are 3nethra Classic, Canon CR-1 Mark II, Nidek AFC-230, Topcon NW300, and EasyScan. The 3nethra Classic (Forus, India) provides color fundus images with size of 2048 × 1536, and the FOV is 40 degrees. The Canon CR-1 mark II (Canon, Japan) is a nonmydriatic retinal camera with FOV of 45 degrees, and the image size is 3456 × 2304. The Nidek AFC-230 (Nidek, Japan) is also a nonmydriatic autofundus camera with 45-degree FOV and captures the fundus on a 3744 × 3744 color image. The Topcon NW300 (Topcon, Japan) is a color fundus camera with picture angle of 45 degrees and its image size is 2048 × 1536. Finally, EasyScan (iOptics, Netherlands) is a scanning laser ophthalmoscopy (SLO) camera with FOV of 45 degrees and the image size of 1024 × 1024.

## 3. Methodology

**Figure 1:**
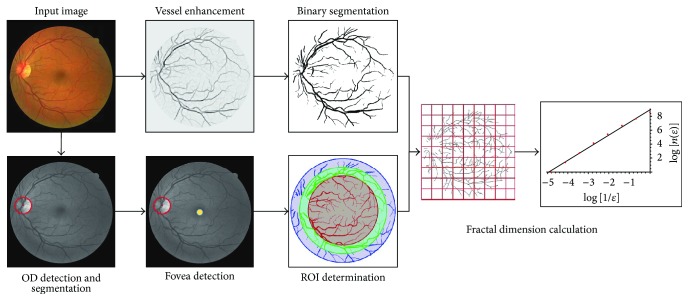
The pipeline for calculating the fractal dimension from a color fundus image.

In this section, we introduce the pipeline and methodologies, which are used to compute the fractal dimension from a fundus image. The pipeline involves 6 steps (see Figure 1). First of all, we import the raw color images from each dataset and rescale them to the same pixel size as the images in the DRIVE dataset. As a result of the acquisition process, very often the retinal images are nonuniformly illuminated and exhibit local luminosity and contrast variability. In order to overcome this problem, each image is preprocessed using the method proposed by Foracchia et al. [23], which normalizes both luminosity and contrast based on a model of the observed image. Luminosity and contrast variability in the background are estimated and then used for normalizing the whole image.

After the image local normalization, we apply 3 state-of-the-art vessel segmentation methods on color retinal images and one particular segmentation method on the SLO images to obtain the vessel probability maps (soft segmentation). Afterwards, a threshold value is applied to the obtained vessel probability maps in order to construct binary segmentations (hard segmentations). At the same time, we automatically determine the region of interest for FD calculation by detecting, segmenting, and parameterizing the optic disc and the fovea. Finally, the fractal dimension is calculated on the binary vessel segmented images within a circular ROI using 3 classic FD measurements. In the following section, each step of the pipeline is introduced in detail.

### 3.1. Automatic Vessel Segmentation Methods

The fractal dimension is usually calculated on a vessel binary map, where pixel intensity of 1 is considered as vessel and 0 as background. Generally manual vessel annotations provided by the human observers have better quality than automatic vessel segmentation techniques. Additionally, for large volume clinical studies, an automatic vessel segmentation program is needed for the vessel detection. In our study, we investigated three vessel segmentation methods for extracting the vessels from RGB retinal images, Frangi's vesselness method, Soares' method, and Zhang's method, and the BIMSO method for SLO images.

#### 3.1.1. Frangi's Vesselness

**Figure 2:**
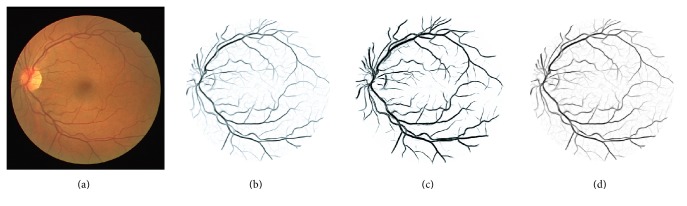
(a) An original image from the DRIVE database; (b)–(d) the vessel probability maps generated by the methods of Frangi et al. [18], Soares et al. [19], and Zhang et al. [20].

 Frangi's vesselness is a multiscale vessel enhancement method proposed by Frangi et al. [18], which uses the second-order derivatives to enhance elongated structures in the image. An important property for an elongated structure is a large change of gradient (principal curvature) in one direction but little gradient change in the direction perpendicular to the former. Therefore, the pixels of a vessel have *λ*_1_⩾*λ*_2_, where *λ*_1_ and *λ*_2_ are the magnitudes of the local principle curvatures that can be calculated via the eigenvalues of the 2D Gaussian Hessian. Thus, the vessels can be enhanced by a normal probability distribution function:

(1) $$\begin{array}{c} \exp\left(\;-\frac{R_{A}^{2}}{2\alpha^{2}}\;\right)\left(\;1-\exp\left(\;-\frac{S^{2}}{2\beta^{2}}\;\right)\;\right), \end{array}$$

where *λ*_1_ and *λ*_2_ are the eigenvalues of the 2D Gaussian Hessian, *R*_*A*_ = *λ*_2_/*λ*_1_ is an anisotropy (elongatedness) term, *S* = *λ*_1_ + *λ*_2_ is a structure term, and *α* and *β* are constant values that determine the sharpness of the filter. The vessel probability map generated by this method is shown in Figure 2(b).

#### 3.1.2. Soares' Segmentation

Soares' segmentation is a supervised method for vessel enhancement proposed by Soares et al. [19]. First it extracts 5 features including the pixel intensity (the green channel) and 4 Gabor filter responses from the images. By using a bank of Gabor filters with multiscales, multifrequencies, and multiorientations, vessels with different sizes and orientations are enhanced and differentiated from the image background.

Afterwards a supervised Gaussian Mixture Model (GMM) classification method is used to classify the pixels into vessel or background using the obtained features. The output is a probability map indicating the likelihood for a pixel being a vessel (shown in Figure 2(c)).

#### 3.1.3. Zhang's Method

Zhang's method is based on describing the image as a function on an extended space of positions and orientations [20]. In the method, the image is lifted to the 3D space of positions and orientations via a wavelet-type transform. In the 3D domain, vessels are disentangled at crossings due to their difference in orientation. In the new space, left-invariant Gaussian derivatives are used (exploiting a rotating coordinate system) in order to enhance the blood vessels. The method results in crossing preserving enhancement of blood vessels (shown in Figure 2(d)).

#### 3.1.4. BIMSO Method

BIMSO method is a brain-inspired multiscale and multiorientation technique proposed by Abbasi-Sureshjani et al. [24], which is mainly designed for the vessel segmentation in SLO images. In this method, the image is lifted to the 3D orientation score using rotated anisotropic wavelets and then a nonlinear transform is applied to enhance the elongated structures (blood vessels) and to suppress the noise. Afterwards, several features for each pixel are extracted including the intensity, filter response to the oriented wavelets, and the multiscale left-invariant Gaussian derivatives jet. The pixels are then classified by a neural network classifier into vessel or background using the obtained features.

### 3.2. Region of Interest (ROI)

In this subtask, the fractal dimensions were calculated in different circular regions with various radii around the fovea and optic disc (OD) centers. For fovea centered images, the regions of interest were centered at the fovea centralis with radii of 4, 5, and 6 times the optic disc radius (OD_*r*_). These radii were set in accordance to the diameter of human optic discs and the average fovea-to-disc distance. According to the study of [25], the average diameter of the human optic disc is 1.83 mm and the distance from the fovea center to the optic disc center is 4.93 mm, which is about 5 times OD_*r*_. Therefore, the circular ROI with radius 4 × OD_*r*_ covers the retina but excludes the optic disc, the 5 × OD_*r*_ ROI covers half of the optic disc, and the 6 × OD_*r*_ ROI covers the full optic disc. Throughout the studies, the ROI is determined automatically by a pipeline described in the following subsubsections.

#### 3.2.1. Optic Disk Detection

Optic Disk detection is done using the method proposed by Bekkers et al. [26]. In this method, the OD is detected via a cross-correlation based template matching in higher dimensional objects called orientation scores. An orientation score represents image data on the 3D space of positions and orientations, where the vessels with different orientations are lifted to different planes of the space. The templates are designed to detect the 3D pattern of vessels originating from the optic nerve head. Therefore, the global maximum of the correlated image reveals the position of the OD.

#### 3.2.2. Optic Disk Segmentation

**Figure 3:**
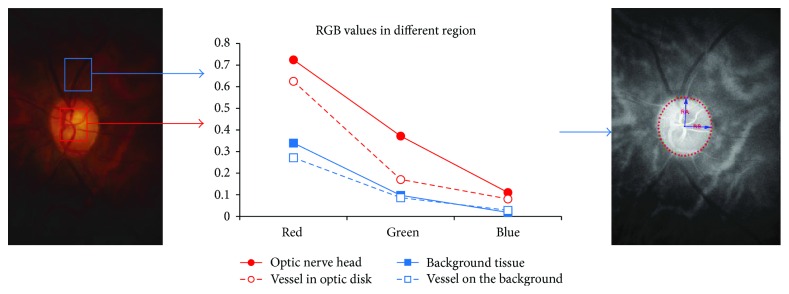
The RGB color difference between the pixels inside and outside the optic nerve head region.

Optic disk segmentation is performed after locating the OD centralis. The segmentation is done within a small patch of an enhanced OD to detect its circular boundary. On a regular RGB fundus image, the OD region has higher color differences than the background region. For instance, the tissue and vessels inside the disc have greater yellow-blue color difference than the background vessel and tissue (see Figure 3). Therefore, the color derivatives of the red, green, and blue intensity can be used to enhance the OD region and suppress the background tissue of the retina.

The color derivatives of an RGB image are computed using the Gaussian color model proposed in [27, 28], where the best linear transform from the RGB color domain to the Gaussian color model is defined by

(2) $$\begin{array}{c} \left[\begin{array}{c} e\\ e_{\lambda }\\ e_{\lambda \lambda } \end{array}\right]=\left(\begin{array}{ccc} 0.06 & 0.63 & 0.31\\ 0.19 & 0.18 & -0.37\\ 0.22 & -0.44 & 0.06 \end{array}\right)\left[\begin{array}{c} \text{R}\\ \text{G}\\ \text{B} \end{array}\right], \end{array}$$

where *e*, *e*_*λ*_, and *e*_*λλ*_ represent the nonderivative, 1st-order derivative, and 2nd-order derivative with respect to the wavelength *λ*. The enhanced OD image is obtained by combining invariant assemblies of *e*, *e*_*λ*_, and *e*_*λλ*_.

After the enhancement, the OD boundary becomes stronger and the potential interferences caused by the edge of vessels are suppressed and a simple zero crossings of the Laplace operator is used for OD edge detection. After that, an ellipse is fitted to the detected boundary positions and the major and minor radius are obtained. Finally, the OD radius (OD_*r*_) is estimated as the average of the major and minor radii of the fitted ellipse.

#### 3.2.3. Fovea Center Detection

Fovea center detection is done within a ring area around the optic disc center. As mentioned earlier, the average distance between the fovea centralis and the optic disc centralis is about 5 × OD_*r*_, so the inner and outer radii of the ring of interest are selected as 4 × OD_*r*_ and 6 × OD_*r*_, respectively. After determining the ring area, we reduced the interference of blood vessels by using the binary vessel segmentation obtained beforehand and an inpainting algorithm which replaces/paints the detected vessels by their neighbor background texture. Finally, the fovea center is detected as the global minimum at a large Gaussian blurring scale.

### 3.3. Fractal Dimension Measurements

The fractal dimension is a measurement which quantifies the highly irregular shape of fractals or fractal objects. An important property of the fractal objects is their self-similarity over different scales or magnifications. This means that at different scales a same pattern with different sizes can be observed, such as trees, snowflakes, and river systems. This self-similar property can be described by the following formula:

(3) $$\begin{array}{c} N\left(\;r\;\right)=r^{-D}, \end{array}$$

where *N*(*r*) is some measurements applied on the complicated pattern of the object at a scale *r*; *D* is the fractal dimension that implies how many new similar patterns are observed as the resolution magnification (scale) decreases or increases. In order to solve for *D* we rewrite (3) into

(4) $$\begin{array}{c} D=-\frac{\log N\left(r\right)}{\log r}. \end{array}$$

According to the definition, a fractal object is self-similar; therefore the comparison of two measurements in various scales should yield the same results. This implies that the fractal can also be calculated by comparing the measurements between any two scales:

(5) $$\begin{array}{c} D\approx -\frac{\log N\left(r_{n}\right)-\log N\left(r_{n-1}\right)}{\log r_{n}-\log r_{n-1}}. \end{array}$$

Based on the above relation between measurements in different scales, a box-counting method is introduced to do a simple, fast estimation of the fractal dimension *D*. In this method, the full space is firstly covered by squared boxes with side-length *r*_*n*_. And then measurements are done in the boxes that are overlapping with the objects. This step will be repeated multiple times with different box side-lengths. Finally, the size of the box and the corresponding measurement are plotted in a log-log plot. The estimated fractal dimension is the slope of the regression line that fits to these data points.

In this paper, we are mainly interested in three fractal methods that are widely used in the literature: the box dimension *D*_*B*_, information dimension *D*_*I*_, and correlation dimension *D*_*C*_, which measure different properties (different *N*(*r*)) of the self-similar pattern of the object, respectively.

#### 3.3.1. Box Dimension (*D*_*B*_)

Box dimension (*D*_*B*_) is the most simple and popular method for estimating the FD of fractal objects proposed by [29]. It is the direct implementation of the Hausdorff dimension in mathematics [30]. The box dimension is defined as the real number *D*_*B*_, such that the number *N*(*r*) of balls with radius *r* that is needed to cover an object grows with (1/*r*)^*D*_*B*_^ as *r* → 0. In other words, *D*_*B*_ is calculated via

(6) $$\begin{array}{c} D_{B}=\underset{r\to 0}{\lim}\frac{\log N\left(r\right)}{\log1/r}. \end{array}$$

So, in the image domain, the measurement *N*(*r*) in (6) is the number of boxes with side-length *r* which overlap with the vessel segmentation. When dealing with discrete problems, taking the limit *r* → 0 is not possible. Instead, as suggested by [29], *D*_*B*_ can be computed as the slope of *N*(*r*) plotted against *r* in a log-log plot.

#### 3.3.2. Information Dimension (*D*_*I*_)

Information Dimension (*D*_*I*_) is inspired from information theory. In information theory, entropy is the measure of the uncertainty of a random event. The less likely a random event might happen, the more informative it is and thus the larger entropy it has. Conversely, if an event happens very often, it provides less information, implying lower entropy. The information dimension [31, 32] is defined as

(7) $$\begin{array}{c} D_{I}=\underset{\delta \to 0}{\lim}\frac{\sum_{i=1}^{N}p_{i}\log p_{i}}{\log1/\delta }, \end{array}$$

where *N* is the number of boxes with size *δ* overlapped with the object, the numerator ∑_*i*=1_^*N*^ *p*_*i*_log⁡*p*_*i*_ is the first-order Shannon entropy, *p*_*i*_ = *n*_*i*_/*M* is the probability for finding a part of the object in the *i*th box, *M* is the total mass of it, and *n*_*i*_ is the part of the object in the box. The limit of (7) is estimated as the slope of the regression line of the logarithmic points.

#### 3.3.3. Correlation Dimension (*D*_*C*_)

Correlation dimension (*D*_*C*_) estimates the FD via the relationship between two pixels inside a region. A correlation integral is defined via the Heaviside step function for counting the pair of points in a region with size *r*_*k*_ and can be approximately expressed in terms of the probability density:

(8) $$\begin{array}{c} C_{k}=\frac{1}{N^{2}}\overset{N}{\underset{\begin{array}{c} i=1,j=1,i\neq j \end{array}}{\sum }}\Theta \left(\;r_{k}-\left\|\;x_{i}-x_{j}\;\right\|\;\right)\approx \overset{N_{k}}{\underset{j=1}{\sum }}p_{jk}^{2}, \end{array}$$

where Θ(*x*) is the Heaviside step function, **x**_*i*_ is the *i*th pixel belonging to an object, and *p*_*jk*_ = *n*_*jk*_/*M* is the probability density of the object with mass *M* in the *j*th box with size *r*_*k*_. The correlation dimension *D*_*C*_ is defined via the relationship between *C*_*k*_ and *r*_*k*_ as *D*_*C*_ = lim_*r*_*k*_→0_(log⁡*C*_*k*_/log⁡*r*_*k*_).

## 4. Stability Analysis and Results

In this section, we present our stability analysis of the fractal methods in terms of the choice of manual annotations, different segmentation methods, various regions of interest, the accuracy of the segmentation method, and different imaging modalities. To study the variation of FDs, we use the relative error (RE) with respect to the binary images annotated by Observer 1 as the reference. The RE is obtained using |(*D*_*x*_ − *D*_*r*_)|/*D*_*r*_, where *D*_*x*_ is the obtained FD in different studies and *D*_*r*_ is the reference FD. To test whether or not measurement methods are correlated, we use the Pearson correlation coefficient test.

*Study 1: Intergroup and Intragroup Fractal Dimension Variation*. In order to show the significance of these relative errors in different experiments, the obtained FD values are compared with the coefficient of variation, also known as relative standard deviations (RSD) of all subjects in the DRIVE dataset, which are 2.3%, 2.1%, and 2.0% for *D*_*B*_, *D*_*I*_, and *D*_*C*_, respectively.

**Figure 4:**
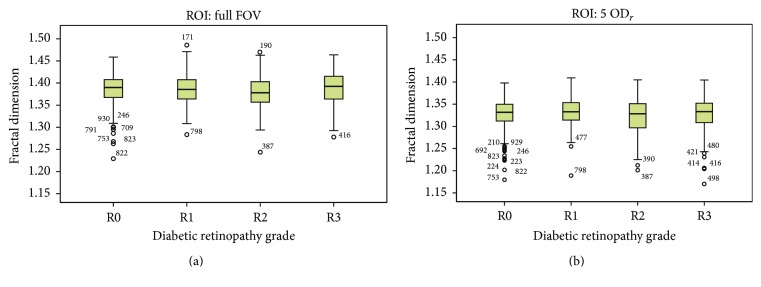
Box plots of the fractal dimensions (*D*_*B*_) of different DR groups for (a) ROI: full FOV and (b) ROI: 5 × OD_*r*_.

We also obtained the intergroup and intragroup fractal dimension (*D*_*B*_) variations for the different groups of diabetic retinopathy in the MESSIDOR dataset. For all images with different DR grades, the box dimension is calculated once on the full image and once inside the region of interest around the fovea (5 × OD_*r*_). The averages and relative standard deviations of FD values for each separate DR group are shown in Table 1. As we can see in this table and in Figure 4, the differences between the mean of FD values for different DR groups are small compared to the RSD of each DR group. The average of RSD in the different groups is higher than 2.5%.

The results of multiple one-way ANOVA tests are shown in Table 2. With this test, we study whether a pair of subgroups have different distributions. In the case of using the full FOV as ROI, there are no significant mean differences between any two groups, except in group pairs R0–R2 and R2–R3. For the circle ROI around the fovea, the mean difference is significant between R0 and R2 and between R1 and R2 groups.

*Study 2: Variation between Different Manual Annotations*. We compared the FD values that were calculated on the ground truth images annotated by two experts within the circular ROI with 5 × OD_*r*_. Here we used the FDs of Observer 1 as reference as this is also considered as ground truth in [22]. The result is shown in the 1st row of Table 3. The main difference between the two manual annotations is the presence of the tiny vessels. We found that missdetecting the tiny vessels does affect the fractal dimension. The maximal differences of 7.11%, 6.70%, and 6.23% and mean relative errors of 1.97%, 1.88%, and 1.77% are obtained for *D*_*B*_,  *D*_*I*_, and *D*_*C*_, respectively, which are noticeable compared to the calculated RSDs.

**Figure 5:**
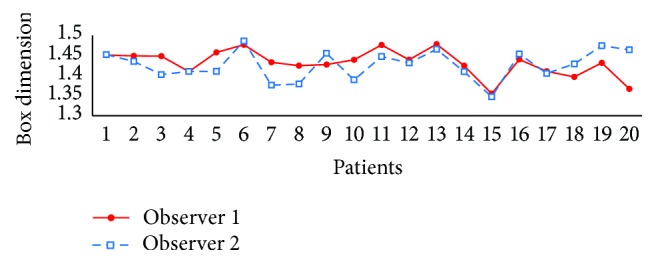
The box dimension values using the manual segmentation by two observers for all patients.

It means that even if the FDs are calculated on vessel maps annotated by human observers, the methods cannot produce stable values for diagnosis, which makes fractal dimension measurement useless. In addition, Figure 5 plots *D*_*B*_ of 20 images of the two observers. The curves illustrate that the variations of FD for two observers in some subjects are too large which might cause wrong discrimination among subjects for clinical applications. For example, we see *D*_*B*_ of patient 5 is greater than patient 4 for Observer 2, while the two patients have similar values obtained from the other observer.

*Study 3: Variation between Different Vessel Segmentation Methods*. In this study, we investigated the variation of fractal dimensions when using automatic vessel segmentation methods instead of human annotations. The methods by Frangi et al. [18], Soares et al. [19], and Zhang et al. [20] were used as described previously. Each method produces a vessel probability map from the raw fundus image from which we obtain a binary map by setting an optimal threshold. The optimal threshold for each method is set to the value which maximizes the vessel segmentation accuracy for the whole dataset. For measuring the errors, we used the FDs of Observer 1 as reference. The 2nd to 4th rows of Table 3 show the REs when using the binary images created by the segmentation methods instead of human observers.

**Figure 6:**
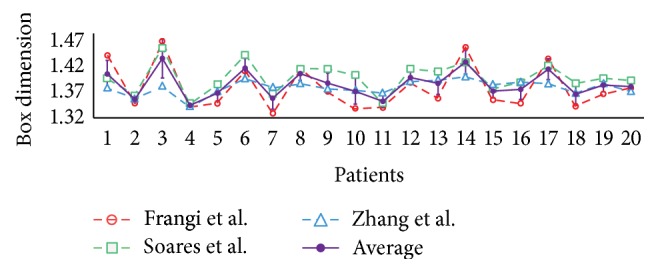
The box dimension values using different segmentation methods for all patients.

The maximum errors of the box dimension for the three segmentation techniques are 9.32%, 8.70%, and 7.37%, respectively. The average errors are 4.29%, 2.88%, and 3.97%, which are significantly compared to the RSD values. These values suggest that using an automatic segmentation would induce a large error in fractal calculation. In addition, the very high *p* values imply the weak association between the automatic methods and the manual. The variation among different segmentation methods is also large according to curves shown in Figure 6, which shows the mean and standard deviation of *D*_*B*_ among the 3 methods. This suggests that the fractal measurement is very sensitive to the choice of vessel segmentation method.

**Figure 7:**
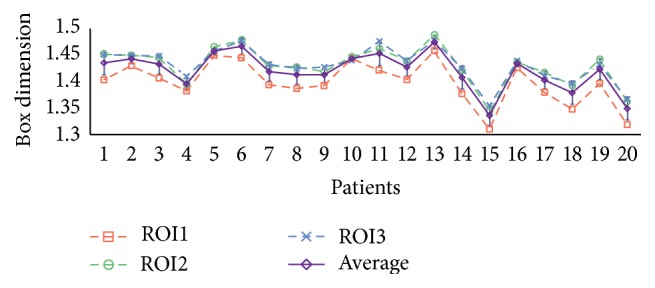
*D*_*B*_ of 20 subjects varied with the change of the ROI.

*Study 4: Different Regions of Interest*. We calculate the FD in various circular regions around the fovea center of the DRIVE ground truth images annotated by Observer 1. As mentioned previously, the ROI radii are considered as 4 × OD_*r*_ (ROI1), 5 × OD_*r*_ (ROI2), and 6 × OD_*r*_ (ROI3), and ROI3 is used as reference for the relative error calculation. The relative errors of changing the ROI are shown in Table 4. When FDs are calculated in ROI1, the maximum error of the box dimension is 3.8%, and the average error is 2.4%. If we use ROI2, the relative errors were smaller, with a maximum of 1.0% and average of 0.4% error.  Figure 7 shows the plot of *D*_*B*_ calculated in ROI1 (red), ROI2 (green), and ROI3 (blue) and also the mean and deviation of them (purple). According to the table and figure, changing ROI causes a variation in fractal calculation; in particular the FDs of ROI1 are significantly lower compared to ROI2 and ROI3. But, from another point of view, we see that *p* values are less than 0.01, which means the FDs calculated in different ROIs are significantly associated.

*Study 5: Vessel Segmentation Method Quality*. We studied the relation between the FD error and the quality of vessel segmentation methods. The FD is usually calculated on a vessel binary map, which is converted from the vessel probability map with a threshold value. The choice for threshold value changes the accuracy of vessel segmentation, and the accuracy of the segmentation method turns out to affect the fractal measurement significantly. The comparison is based on Zhang's segmentation method in a fixed region of interest (ROI2). Several threshold values *t* with range from 0.15 to 0.35 and step size 0.01 are applied to the vessel probability map for all test images in the DRIVE database to obtain the vessel binary segmentations. Since there is a large difference between number of vessel pixels and nonvessel pixels in retinal images, we used the Matthews correlation coefficient (MCC) instead of accuracy to evaluate the quality of binary images. The MCC is a balanced measure which can be used even if the classes are of very different sizes:

(9) MCC=TP×TN−FP×FNTP+FPTP+FNTN+FPTN+FN,

where TP, TN, FP, and FN are the true positive, true negative, false positive, and false negative parts of the segmentation with respect to the annotations by Observer 1. For each result of the binary segmentation, the fractal dimension is measured and compared to the values of the reference ones.

**Figure 8:**
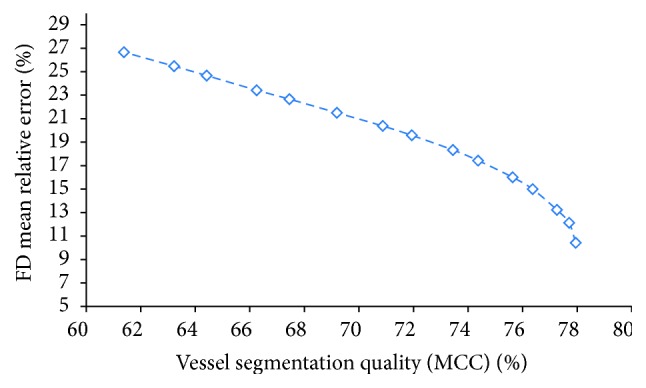
The mean relative error of fractal dimension against the quality of vessel segmentation based on MCC.

The mean relative errors for the 20 images with respect to the reference ones are shown in Table 5 for 3 sample thresholds* t* = 0.15, 0.21, and 0.34. As we can see in this table, using both 0.15 and 0.34 as threshold results in similar MCC values for the vessel segmentation, while one is the oversegmented (higher FD) and the other one is the undersegmented (lower FD), a threshold equal to 0.21 gives the highest MCC 78% as an average among 20 images. Note that no postprocessing was applied after the thresholding, so the segmentation accuracy in our studies might be lower than the proposed accuracy in the literature. From the table, we see that if the threshold is set properly (*t* = 0.21), the relative error is small. Meanwhile if *t* is underestimated or overestimated, the relative error dramatically increases. Moreover, Figure 8 shows the plot of the mean MCC of vessel segmentation against the mean error of FD of 20 images. We can see that segmentation with higher accuracy produces a more reliable FD. These results suggest that poor segmentation with improper selection of the threshold value leads to a large error for fractal dimension calculation.

**Figure 9:**
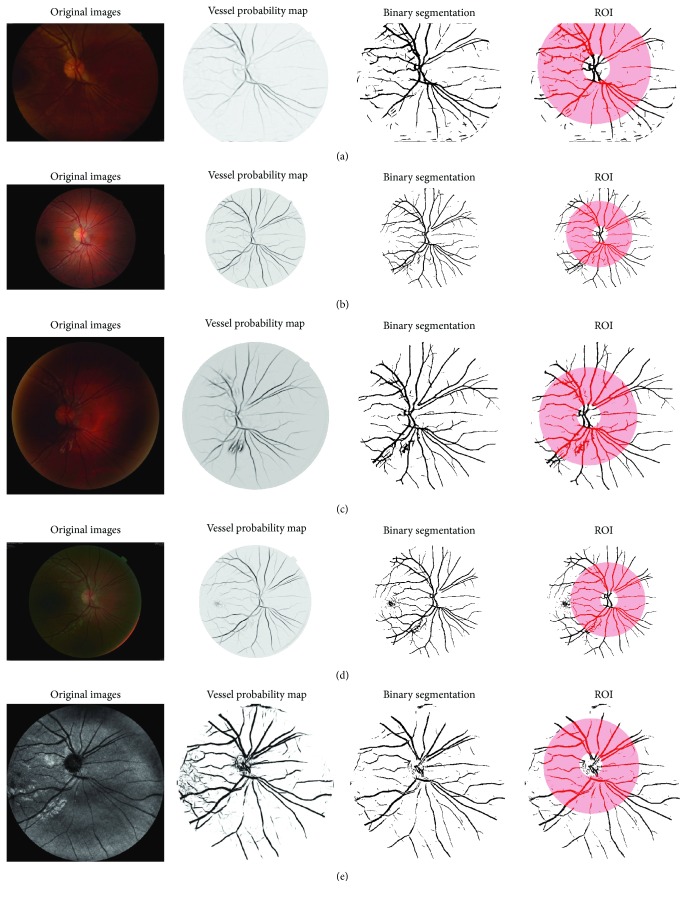
The retina of one subject captured by different cameras: (a) 3nethra, (b) Canon, (c) Nidek, (d) Topcon, and (e) EasyScan.

*Study 6: Different Cameras and Different Image Modalities*. We investigated the variation of fractal dimension which is calculated on the images captured by different cameras described previously. The optic disc centered images of the 12 volunteers are used in this examination (see Figure 9). The circular region of interest centered at the OD center with radius 4 × OD_*r*_ is used in all images. The vessel segmentation results of the RGB images captured by regular cameras are generated by Zhang's [20] method and those of the EasyScan SLO camera are generated by the BIMSO method [24].

**Figure 10:**
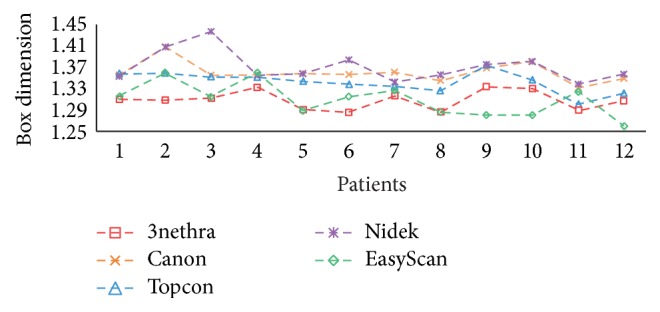
*D*_*B*_ calculated on the images captured by 5 different fundus cameras.

First we compare the variation among different cameras, where the box dimensions of 12 subjects are shown in Figure 10 with different colors per camera. As we can see from this figure, the fractal dimension is very sensitive to image properties like resolution, amount of noise, quality, and imaging modality, which depend on the type of camera. For example, the mean relative difference between 3nethra (red dashed line) and Nidek (purple dashed line) is 2.31% with respect to the average of two cameras. Moreover, using different imaging modalities also causes a significant variation. The SLO images acquired by EasyScan (green dashed line) in general have lower FDs than the other color RGB cameras except for 3nethra. In addition, the average relative variation between the SLO images and RGB images (by Canon camera) is 1.95%.

Finally, we investigate the repeatability of different cameras by comparing the FDs of different acquisitions of one subject. The repeatability is measured as the standard deviation of the fractals calculated on 5 acquisitions of the same subject divided by the average of them. As we can see from Table 6, the 5 cameras give an average of 1.11% variation on the same subject in different acquisition times. With Canon and Nidek cameras, this error is small (0.69% and 0.96% resp.), which shows better stability compared to other cameras.

## 5. Discussion

In previous studies, fractal dimension is considered as a potential biomarker for disease detection. However, conflicting findings were found in different literature. Therefore, we examined the reliability of three classic fractal measurements for their use in clinical study applications. We divided our experiments into six studies, which we will discuss in the remainder of this section.

**Figure 11:**
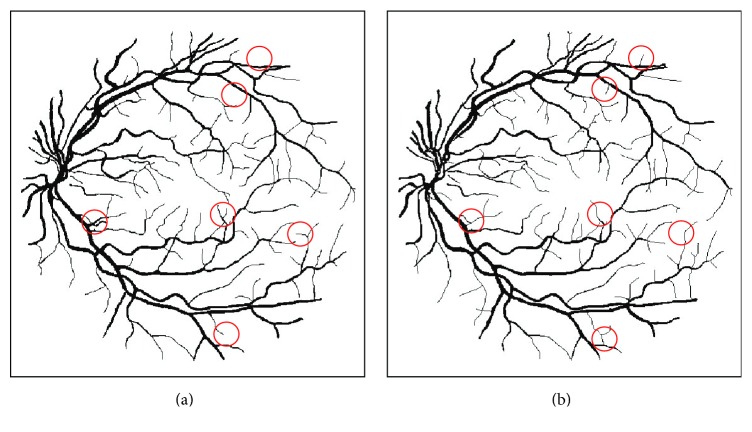
The vessel annotations of 2 human observers. The major difference is the missing of small vessels, as indicated by the red circles; (a) Observer 1 (*D*_*B*_ = 1.468) and (b) Observer 2 (*D*_*B*_ = 1.450).

In our first and second studies, we investigated intergroup and intragroup variability of FD methods using the MESSIDOR dataset. Also, we studied intraobserver variation using ground truth segmentation from the DRIVE dataset. The experimental results show that, even with ground truth vessel maps, the fractal dimensions are not reliable. The RSD of *D*_*B*_ of all patients in the DRIVE dataset is 2.3%. Moreover, the variation of FD between different human observers produces errors of 1.97%, 1.88%, and 1.77% on average on *D*_*B*_, *D*_*I*_, and *D*_*C*_. This significant variation makes the FD less informative and less reliable in discriminating DR patients in different severity levels from the healthy ones. No significant differences in FD were found between different DR groups of the MESSIDOR dataset. From Figure 11, we see that the main difference between the vessel annotations of two observers is the presence or absence of small vessels. Therefore, the influence of small vessels on the fractal measurements cannot be neglected and should be considered seriously.

In the third study, we investigated the influence of automatic segmentation method on FD computations. We examined the FD on the vessel maps produced by three different vessel segmentation methods on the same imaging modality (RGB fundus images). The results show that the FDs calculated with various segmentations have significant differences compared to the values calculated using the annotations by Observer 1. In addition, the statistical tests show that the FDs were not associated with those computed from ground truth images. Therefore, the FD computed by automatic computer software might not be reliable, as was the case in the studies from [4–8].

In the fourth study, we investigated the variation of FD calculated within different regions of interest centered at the fovea centralis. This study is motivated by the fact that, in clinical retinal photography, the actual captured area on the retina is not always the same because of eye motion. The result shows that FDs calculated in 3 different ROIs are associated with each other, with *p* values less than 0.01. However, as we can see from Figure 7, a smaller ROI produces a lower FD in general, because fewer vessels are taken into account. Therefore, this study implies that a fixed region of interest is necessary in order to obtain comparable FD values.

In the fifth study, we investigated the influence of the accuracy of vessel segmentation methods on the fractal measurements. Most vessel segmentation methods need a threshold value to convert the vessel probability map into a vessel binary map. This threshold value also affects the accuracy of the segmentation. In this study, we computed the FD on vessel binary segmentations using different thresholds (MCC ranged from 61% to 78%). As expected, the computed FD values become closer to the ones obtained from manual segmentations when segmentation accuracies increase (with respect to manual segmentation). Moreover, the variation decreases faster when the segmentation accuracy is higher than 75%. Therefore, a proper thresholding technique is required to obtain a stable FD measurement.

Finally, in the sixth study, we compared the FDs calculated on images acquired by different fundus cameras. The result shows that the variations of FD are significant when different cameras are used. These five cameras use different flashing systems resulting in different contrast and tissue reflections. Finally, the image sizes and resolutions are different, so the details of retina captured by these cameras are also not identical. Moreover, some cameras were easier to operate (e.g., via autofocus), resulting in more consistent image quality. The comparison result shows that, in general, the FD of the same subject using different cameras has significant differences. The differences in terms of image properties cause significant variations as we see from the results.

Besides the variation between cameras, we also investigated the repeatability of the FD measurement on the same subject using the same camera. The slight differences among multiple acquisitions on the same patient with the same camera are caused by variation in image quality, for example, caused by eye motions (blurry image), weak flashing/illumination, or incorrect focusing. The results show that the 5 cameras generally produce 1.11% variation between multiple photographs.

## 6. Conclusion

Our experiments suggest that the classic fractal dimensions must be calculated under very strict conditions, and tiny changes on the images and vessel segmentation can cause significant variations. The vessel segmentation method must be very carefully chosen, the region of interest in all images must be equally set for the FD calculation, and an optimal threshold value for creating a high accuracy binary vessel segmentation map is required. For future studies, FD's high sensitivity to the segmentation methods and thresholding techniques will be addressed by measuring FD directly from the vessel probability maps.

## Figures and Tables

**Table 1:** The mean and standard deviation of FD values (*D*_*B*_) for different DR grades.

DR grade	Number of images	ROI: full FOV			ROI: 5 × OD_*r*_		
		Mean	SD^*∗*^	RSD^†^	Mean	SD	RSD
R0	546	1.3864	0.0324	2.34%	1.3285	0.0316	2.38%
R1	153	1.3852	0.0345	2.49%	1.3317	0.0304	2.28%
R2	247	1.3781	0.0364	2.64%	1.3215	0.0384	2.91%
R3	254	1.3869	0.0384	2.77%	1.3276	0.0375	2.82%

Total	1200	1.3846	0.0350	2.52%	1.3273	0.0343	2.59%

^*∗*^SD: standard deviation.

^†^RSD: relative standard deviation.

**Table 2:** Comparison between FD values in different DR groups (ANOVA test).

DR grade			Mean difference	Std. error	*p* value^†^	95% confidence interval	
						Lower bound	Upper bound
ROI: full FOV	R0	R1	0.00123	0.00319	0.981	−0.0070	0.0094
		R2	0.00834^*∗*^	0.00267	**0.010**	0.0015	0.0152
		R3	−0.00046	0.00265	0.998	−0.0073	0.0063

ROI: full FOV	R1	R2	0.00711	0.00358	0.195	−0.0021	0.0163
		R3	−0.00169	0.00356	0.965	−0.0109	0.0075

ROI: full FOV	R2	R3	−0.00879^*∗*^	0.00311	**0.025**	−0.0168	−0.0008

ROI: 5 × OD_*r*_	R0	R1	−0.00324	0.00313	0.730	−0.0113	0.0048
		R2	0.00696^*∗*^	0.00263	**0.040**	0.0002	0.0137
		R3	0.00086	0.00260	0.987	−0.0058	0.0076

ROI: 5 × OD_*r*_	R1	R2	0.01020^*∗*^	0.00352	**0.020**	0.0011	0.0193
		R3	0.00410	0.00350	0.646	−0.0049	0.0131

ROI: 5 × OD_*r*_	R2	R3	−0.00610	0.00306	0.191	−0.0140	0.0018

^*∗*^The mean difference is significant at the 0.05 level.

^†^One-way ANOVA test with null hypothesis that the means of distributions are equal.

**Table 3:** The comparison of FD between two human observers and different vessel segmentation methods by considering Observer 1 as reference.

Method	Box dimension (*D*_*B*_)			Information dimension (*D*_*I*_)			Correlation dimension (*D*_*C*_)		
	Max^*∗*^	MRE^†^	*p* value^‡^	Max	MRE	*p* value	Max	MRE	*p* value
Observer 2	7.1%	2.0%	**0.0585**	6.7%	1.9%	**0.0851**	6.2%	1.8%	**0.0974**
Frangi [18]	9.3%	4.3%	0.8035	9.4%	4.3%	0.8802	9.4%	4.3%	0.6990
Soares [19]	8.7%	2.9%	0.4926	8.7%	3.0%	0.7339	8.9%	3.0%	0.8657
Zhang [20]	7.4%	3.9%	0.4950	7.4%	3.8%	0.8506	7.3%	3.8%	0.691

^*∗*^Max: maximum relative error with respect to Observer 1.

^†^MRE: mean of relative error with respect to Observer 1.

^‡^Pearson correlation test with null hypothesis that the correlation coefficient is zero.

**Table 4:** The comparison of *D*_*B*_ values using different region of interest.

Method	Radius	Max	MRE	*p* value^*∗*^
ROI1	4 × OD_*r*_	3.8%	2.4%	<0.01
ROI2	5 × OD_*r*_	1.0%	0.4%	<0.01
ROI3	6 × OD_*r*_	Reference		

^*∗*^Pearson correlation test with null hypothesis that the population correlation coefficient is zero with respect to ROI3.

**Table 5:** FD variation against vessel segmentation accuracy.

	Threshold		
	*t* = 0.15	*t* = 0.21	*t* = 0.34
Average of vessel segmentation accuracy	63.74%	77.95%	63%
Average of FD variation	14.44%	10.42%	25%

**Table 6:** The mean relative error of FD for repeated acquisitions in different cameras.

Camera	Image modality	Image size	FOV	Max RSD	Mean RSD
3nethra	RGB	2048 × 1536	40°	2.60%	1.25%
Canon	RGB	3456 × 2304	45°	1.64%	0.69%
Topcon	RGB	2048 × 1536	45°	3.86%	1.41%
Nidek	RGB	3744 × 3744	45°	2.20%	0.94%
EasyScan	SLO	1024 × 1024	45°	3.68%	1.25%
Average	—	—	—	2.80%	1.11%
